# Remote Monitoring and Data Collection for Decentralized Clinical Trials

**DOI:** 10.1001/jamanetworkopen.2024.6228

**Published:** 2024-04-12

**Authors:** Bobby Daly, Otis W. Brawley, Mary K. Gospodarowicz, Olufunmilayo I. Olopade, Lola Fashoyin-Aje, Victoria Wolodzko Smart, I-Fen Chang, Craig L. Tendler, Geoffrey Kim, Charles S. Fuchs, Muhammad Shaalan Beg, Lianshan Zhang, Jeffrey J. Legos, Cristina Ortega Duran, Chitkala Kalidas, Jing Qian, Justin Finnegan, Piotr Pilarski, Harriet Keane, Johanna Shen, Amy Silverstein, Yi-Long Wu, Richard Pazdur, Bob T. Li

**Affiliations:** 1 Department of Medicine, Memorial Sloan Kettering Cancer Center and Weill Cornell Medicine, New York, New York; 2 School of Medicine, Department of Epidemiology, Johns Hopkins Bloomberg School of Public Health, Baltimore, Maryland; 3 Princess Margaret Cancer Center, University of Toronto, Toronto, Ontario, Canada; 4 Medicine and Human Genetics, Center for Clinical Cancer Genetics and Global Health, University of Chicago Medical Center, Chicago, Illinois; 5 Oncology Center of Excellence, US Food and Drug Administration, Silver Spring, Maryland; 6 Susan G. Komen Foundation, Dallas, Texas; 7 Amgen Inc, Thousand Oaks, California; 8 Janssen, Johnson & Johnson, New Brunswick, New Jersey; 9 BeiGene, Cambridge, Massachusetts; 10 Genentech, South San Francisco, California; 11 Yale Cancer Center, Yale School of Medicine, New Haven, Connecticut; 12 Science 37, Durham, North Carolina; 13 Internal Medicine, Gastrointestinal Oncology, Simmons Comprehensive Cancer Center, University of Texas Southwestern Medical Center, Dallas, Texas; 14 Jiangsu Hengrui Pharmaceuticals, Shanghai, China; 15 Novartis, Basel, Switzerland; 16 AstraZeneca, Cambridge, United Kingdom; 17 Bayer, Leverkusen, Germany; 18 Asia Society, New York, New York; 19 Bloomberg New Economy, Bloomberg LP, New York, New York; 20 McKinsey Cancer Center, McKinsey & Company, New York, New York; 21 Guangdong Lung Cancer Institute, Chinese Thoracic Oncology Group, Guangdong Provincial People’s Hospital, Guangdong Academy of Medical Sciences, Guangzhou, China

## Abstract

**Importance:**

Less than 5% of patients with cancer enroll in a clinical trial, partly due to financial and logistic burdens, especially among underserved populations. The COVID-19 pandemic marked a substantial shift in the adoption of decentralized trial operations by pharmaceutical companies.

**Objective:**

To assess the current global state of adoption of decentralized trial technologies, understand factors that may be driving or preventing adoption, and highlight aspirations and direction for industry to enable more patient-centric trials.

**Design, Setting, and Participants:**

The Bloomberg New Economy International Cancer Coalition, composed of patient advocacy, industry, government regulator, and academic medical center representatives, developed a survey directed to global biopharmaceutical companies of the coalition from October 1 through December 31, 2022, with a focus on registrational clinical trials. The data for this survey study were analyzed between January 1 and 31, 2023.

**Exposure:**

Adoption of decentralized clinical trial technologies.

**Main Outcomes and Measures:**

The survey measured (1) outcomes of different remote monitoring and data collection technologies on patient centricity, (2) adoption of these technologies in oncology and all therapeutic areas, and (3) barriers and facilitators to adoption using descriptive statistics.

**Results:**

All 8 invited coalition companies completed the survey, representing 33% of the oncology market by revenues in 2021. Across nearly all technologies, adoption in oncology trials lags that of all trials. In the current state, electronic diaries and electronic clinical outcome assessments are the most used technology, with a mean (SD) of 56% (19%) and 51% (29%) adoption for all trials and oncology trials, respectively, whereas visits within local physician networks is the least adopted at a mean (SD) of 12% (18%) and 7% (9%), respectively. Looking forward, the difference between the current and aspired adoption rate in 5 years for oncology is large, with respondents expecting a 40% or greater absolute adoption increase in 8 of the 11 technologies surveyed. Furthermore, digitally enabled recruitment, local imaging capabilities, and local physician networks were identified as technologies that could be most effective for improving patient centricity in the long term.

**Conclusions and Relevance:**

These findings may help to galvanize momentum toward greater adoption of enabling technologies to support a new paradigm of trials that are more accessible, less burdensome, and more inclusive.

## Key Points

**Question:**

What are the current state and future aspirations for the use of remote technologies in oncology clinical trials?

**Findings:**

In this survey study of 8 biopharmaceutical companies representing 33% of the oncology marketplace, the difference between current and aspired adoption of remote technologies in 5 years is large, with respondents expecting a 40% or greater adoption increase in 8 of 11 enabling technologies.

**Meaning:**

These findings set benchmarks that may galvanize momentum toward greater adoption of enabling technologies, supporting a new paradigm of trials that are more accessible.

## Teaser

This survey study assesses the current state of decentralized trial technology adoption and highlights industry aspirations and directions for use to enable more patient-centric trials.

## Introduction

International oncology societies have stated that clinical trials offer the best care for patients with cancer but that less than 5% of patients enroll in trials worldwide.^1^ One cause may be the high financial and logistic burdens of clinical trials, which disproportionately affect underrepresented populations. The disparities may be particularly challenging as most oncology trials are conducted in academic medical centers, but the majority of patients prefer to receive care in the local community.^1,2,3,4^ In the US, nearly one-half of patients with metastatic breast, prostate, colorectal, or non–small cell lung cancer must drive more than 60 minutes each way to access a clinical trial site.^5^ A recent survey indicated that 85% of patients with cancer would be more open to joining a trial where they can participate at local facilities, while 82% indicated that they would participate in trials that used wearable technology.^6^

These trends underscore the opportunity for sponsors (eg, biopharmaceutical companies, academic institutions) and regulators to adopt remote monitoring and data collection in cancer trials to create a more patient-centric experience. This shift would be timely, as regulators in the US, the European Union, and China are all developing formal guidance on decentralized trials.^7,8^ The US Food and Drug Omnibus Reform Act, signed into law in December 2022, includes provisions for modernizing clinical trials and requires the US Food and Drug Administration to issue guidance on decentralized trials, including the use of digital health technologies.^9^ In April 2022, the Food and Drug Administration issued a draft guidance recommending that sponsors submit diversity plans for clinical trials to ensure inclusion of underrepresented populations.^10^ The China National Medical Products Administration’s 2021 draft guideline aims to reduce patient burden to the greatest extent possible during trials without compromising safety or data quality, specifically calling for consideration of telemedicine, wearable medical equipment, and remote research.^11,12,13,14,15^

## Methods

A survey on the current and future adoption of remote monitoring and data collection was developed by the Bloomberg New Economy International Cancer Coalition (the coalition) with the following goals: (1) assess the current state of and future aspirations for industry adoption of remote monitoring and data collection in oncology vs other therapeutic areas and (2) understand the environmental factors and objectives driving or preventing the adoption of remote monitoring and data collection in oncology trials. This survey study was not submitted for institutional review board approval because it did not involve human participant research or health care records. Informed consent to participate in the survey was received verbally from each participant at the time of survey initiation. The survey was drafted with input from select coalition members, excluding survey participants, and there was consideration of the American Association for Public Opinion Research (AAPOR) guidance in planning, designing, and reporting the survey results.^16^

The coalition, established in 2021, represents an international, multistakeholder, private-public collaboration among academia, industry, government, patient advocacy groups, and policy think tanks.^1^ It is dedicated to leveraging technology and fostering synergistic collaborations, with a core aim of enhancing patient access to clinical trials worldwide.

The survey, administered from October 1 through December 31, 2022, focused on registrational clinical trials with questions devoted to all therapeutic areas, including oncology, aligned to the broad goals and mission of the coalition. Global biopharmaceutical companies were invited to participate in the survey based on their membership in the coalition at the time of survey administration. Survey participants were not involved in the data analysis, which was performed between January 1 and 31, 2023. The survey consisted of 7 questions (eMethods in Supplement 1) organized into 3 sections: (1) outcomes of remote monitoring and data collection technologies associated with patient centricity overall and (2) across all therapeutic areas and oncology and (3) context for adoption and tracking.

### Statistical Analysis

The raw data were collected and analyzed using Microsoft Excel, version X (Microsoft Corporation). For adoption rates, the mean and SD were calculated. Effect scores were calculated through assigning answers a number. An answer that ranked the approach as 1 was assigned 3 points, an answer that ranked the approach as 2 was assigned 2 points, and an answer that ranked the approach as 3 was assigned 1 point. Across each answer, the points across the survey respondents were summed.

## Results

All 8 invited companies completed the survey for a 100% response rate. These companies comprise approximately 33% of the global oncology pharmaceutical market by revenue.

### Drivers of Increasing Adoption of Remote Monitoring and Data Collection Technologies

All organizations reported recent increases in adoption of 11 remote monitoring and data collection technologies surveyed (Table). The top 4 reasons for adoption were increased competition for patients in the indication under investigation (6 respondents [75%]), changing expectations on the heels of the COVID-19 pandemic and its associated disruptions (5 respondents [63%]), innovation in novel technical solutions that substantially improved quality and accessibility (5 respondents [63%]), and competition from other stakeholders having encouraged or mandated adoption (5 respondents [63%]). Other reasons included stakeholders (eg, regulatory bodies) encouraging adoption (3 respondents [38%]) (eFigure 1 in Supplement 1).

**Table.** Remote Monitoring and Data Collection Technologies

Technology	Definition
eDiary and eCOA	Electronic methods of capturing notes on patient experience (including adverse events) and efficacy of therapeutics
Patient engagement dashboard	Digital platform with tools and features to facilitate day-to-day trial participation and adherence (eg, patient scheduling, patient reimbursement tracking, symptom assessment, dose reporting)
Digitally enabled enrollment	Methods that support patient enrollment, including prescreening, initial site visit, informed consent, and screening, such as eConsent
Digitally enabled recruitment	Methods that support identification of patients, sourcing, and education of patients for participation in clinical trials, such as digital patient identification and use of social media to identify patients
Remote monitoring	Connected tools and devices to support monitoring of patient health and vitals remotely or outside of a traditional clinical trial site (eg, electrocardiography, pulse oximetry)
Telemedicine visits	Virtual clinical trial visits through use of teleconferencing
Visits in local physician networks	Visits with local oncologists outside the academic trial site (hub-and-spoke network)
Mobile nursing visits	Mobile clinical trial sites that bring health care professionals directly to patients in their homes or places of work
Imaging at sites near patients	Imaging at stand-alone or regional imaging centers
Laboratory data collection near patients	Collection of biospecimens at a retail laboratory or patient’s home
Shipment of medicines to patients’ homes	Delivery using courier services

Abbreviations: eCOA, electronic clinical outcome assessment; eConsent, electronic consent; eDiary, electronic diary.

### Current Adoption of Remote Monitoring and Data Collection Technologies

**Figure 1.**
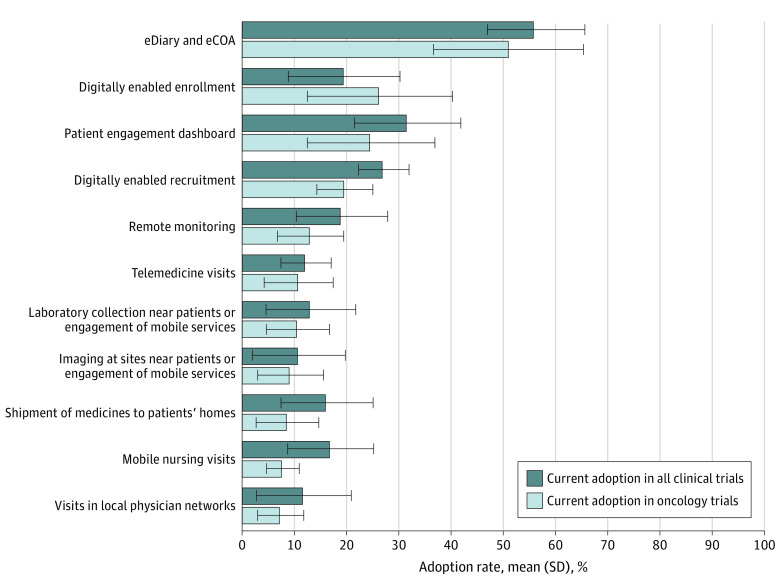
Respondent Adoption Rate of Remote Monitoring and Data Collection Technologies in All Clinical Trials, Including Oncology Trials, Compared With Oncology-Only Trials The data take into consideration adoption in registrational clinical trials only. eCOA indicates electronic clinical outcome assessment; eDiary, electronic diary.

According to the survey, electronic diaries and electronic clinical outcome assessments were the most used technologies, with mean (SD) adoption rates of 56% (19%) and 51% (29%) for all clinical trials and oncology trials, respectively (Figure 1). The next most used technologies were patient engagement dashboards, digitally enabled recruitment, and digitally enabled enrollment, with reported mean (SD) adoption rates of 32% (20%), 27% (10%), and 20% (21%), respectively. The least commonly adopted technologies included permitting patients to use local imaging facilities (mean [SD], 11% [18%] for all trials vs 9% [3%] for oncology trials) or local physician networks (mean [SD], 12% [18%] for all trials vs 7% [9%] for oncology trials), and telemedicine visits (mean [SD], 12% [10%] for all trials vs 11% [13%] for oncology trials) (Figure 1). Adoption rates for remote monitoring and data collection technologies were lower in oncology trials vs all clinical trials for all technologies except digitally enabled enrollment (20% [21%] for all trials vs 26% [28%] for oncology trials).

### Five-Year Aspired Adoption of Remote Monitoring and Data Collection Technologies in Oncology Trials

**Figure 2.**
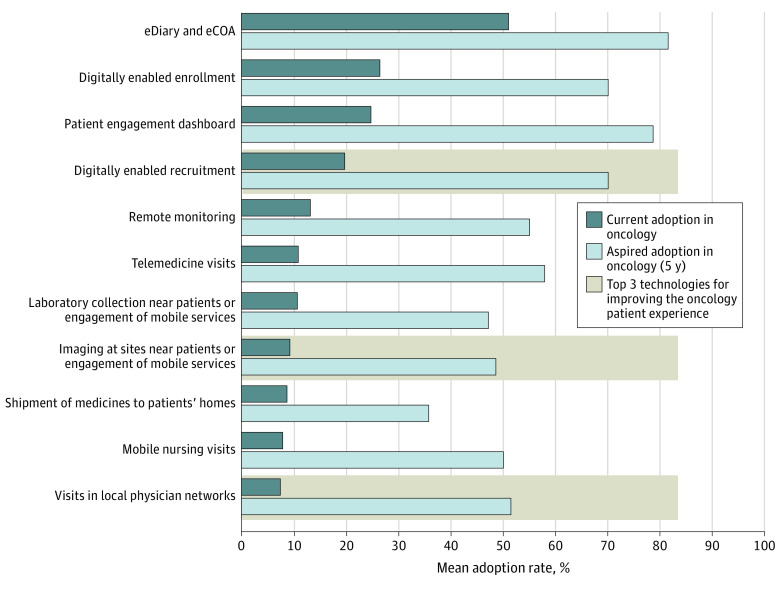
Current Adoption Rate of Remote Monitoring and Data Collection Technologies in Oncology Trials Compared With Average Aspired Adoption Rate in 5 Years The data take into consideration adoption in registrational clinical trials only. eCOA indicates electronic clinical outcome assessment; eDiary, electronic diary.

The difference between current adoption and aspired adoption rates in 5 years of various technologies was large. In 8 of the 11 technologies included in the survey, respondents expected a 40% or greater absolute adoption increase relative to current levels (Figure 2). The greatest mean (SD) differences between current and aspired adoption rates were observed in use of patient engagement dashboards (from 25% [25%] to 79% [20%]), digitally enabled recruitment (from 20% [11%] to 70% [31%]), telemedicine visits (from 11% [13%] to 58% [24%]), visits in local physician networks (from 7% [9%] to 51% [40%]), and digitally enabled enrollment (from 26% [28%] to 70% [26%]).

### Remote Monitoring and Data Collection Technologies With Greatest Outcomes Associated With Patient Experience

Respondents identified telemedicine, digitally enabled recruitment, electronic diaries, and electronic clinical outcome assessments as the most effective technologies for advancing patient centricity within trials (defined in the survey as prioritizing the needs of patients) in the short term (within the next 3 years). Over the long term, respondents identified visits in local physician networks, digitally enabled recruitment, and the use of imaging sites near patients and mobile imaging services as the 3 most effective technologies for improving the oncology patient experience (eFigure 2 in Supplement 1).

## Discussion

In this survey study of global pharmaceutical companies, the results showed that many of the identified remote monitoring and data collection technologies, such as digital enrollment, are already being adopted across clinical trials. Yet adoption of these innovations is lagging in oncology, despite the great unmet need given the relative lack of clinical trial availability in local community settings. While technology advancements have enabled initial adoption of remote monitoring,^17,18^ infrastructure has yet to be built to support clinical trial conduct in local physician networks and imaging facilities in diverse global communities. Such an infrastructure is essential to achieve the greatest long-term effect of decentralized trials.

This survey is the first attempt by a broad coalition of stakeholders with vested interest in advancing patient-centric international cancer trials to set an aspiration for remote monitoring and data collection. The difference between current adoption and aspired adoption rates represents an opportunity to rapidly improve the patient experience in cancer trials, broaden access to clinical trials, and correct historical inequities due to structural barriers, such as lack of access to health care. The Food and Drug Omnibus Reform Act^9^ and recent guidance from regulatory agencies in the European Union^8^ and China^11,12,13,14,15^represent an opportunity to accelerate implementation of technologies with the greatest potential to expand patient access to clinical trials and shorten the timeline for development of innovative cancer therapies and preventive interventions.

### Limitations

This study has some limitations. While survey participants represented 33% of the global oncology pharmaceutical market by revenue, the findings are limited by their membership in the coalition at the time of survey administration and may not be entirely representative of the greater global biopharmaceutical industry.

## Conclusions

The findings suggest that investment may be required across the drug development ecosystem in both technology access and collaborative research infrastructure to equip stakeholders with the capabilities to adopt decentralized technologies in some of the most complex and historically demanding trials. Moreover, for these advances to be successful, solutions are needed to reduce bureaucratic workload for site staff and investigators and not introduce new administrative hardships. The onus is on the oncology industry and research community to ensure that we create opportunities to fund continued advancements; measure our progress; look for and track balancing measures that could compromise progress; and step back regularly to reflect on the net impact on key clinical development objectives, such as cost,^19,20^ quality, speed, experience, and equity.^21,22^

## Notes

Supplement 1. **eFigure 1.** Reasons for Adopting Remote Monitoring and Data Collection Technologies **eFigure 2.** Potential Short-Term (Within 3 Years) and Long-Term (Next 3+ Years) Impact of Remote Engagement and Data Collection Technologies **eMethods.** Bloomberg New Economy International Cancer Coalition–Patient Centricity Survey

Supplement 2.
Data Sharing Statement
