# Supplementary material for: Remote Monitoring and Data Collection for Decentralized Clinical Trials
Source: JAMA Netw Open. 2024 Apr 12;7(4):e246228. doi: 10.1001/jamanetworkopen.2024.6228 (PMC11015350; doi:10.1001/jamanetworkopen.2024.6228)
Supplement: Supplement 1. — eFigure 1. Reasons for Adopting Remote Monitoring and Data Collection Technologies eFigure 2. Potential Short-Term (Within 3 Years) and Long-Term (Next 3+ Years) Impact of Remote Engagement and Data Collection Technologies eMethods. Bloomberg New Economy International Cancer Coalition–Patient Centricity Survey [file jamanetwopen-e246228-s001.pdf]

Supplemental Online Content

Daly B, Brawley OW, Gospodarowicz MK, et al. Remote monitoring and data collection for decentralized clinical trials. *JAMA Netw Open*. 2021;7(4):e246228. doi:10.1001/jamanetworkopen.2024.6228

eFigure 1. Reasons for Adopting Remote Monitoring and Data Collection Technologies

eFigure 2. Potential Short-Term (Within 3 Years) and Long-Term (Next 3+ Years) Impact of Remote Engagement and Data Collection Technologies

eMethods. Bloomberg New Economy International Cancer Coalition–Patient Centricity Survey

This supplemental material has been provided by the authors to give readers additional information about their work.

© 2024 Daly B et al. *JAMA Network Open.*

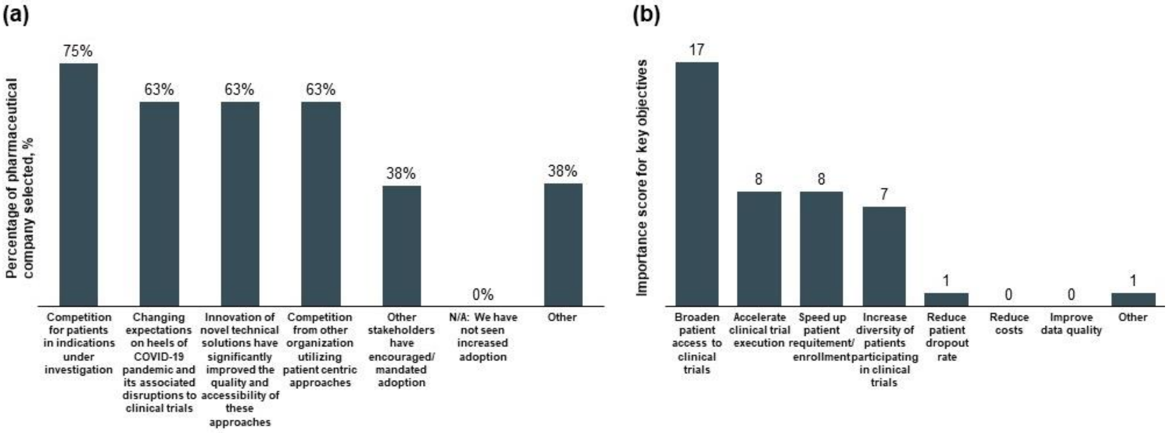

eFigure 1. Reasons for Adopting Remote Monitoring and Data Collection Technologies. (a) Key changes driving increased adoption of remote engagement and data collection approaches. (b) Key objectives for adoption of remote engagement and data collection approaches. Full importance score is 24.

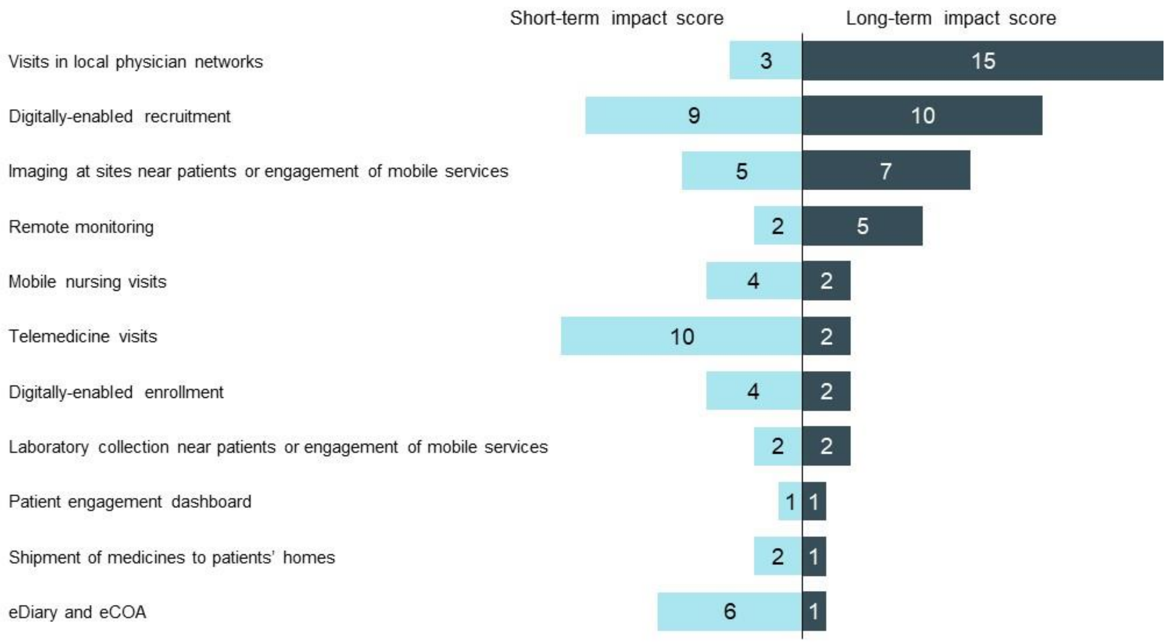

eFigure 2. Potential Short-Term (Within 3 Years) and Long-Term (Next 3+ Years) Impact of Remote Engagement and Data Collection Technologies. Expected improvement in the oncology patient experience. Full score is 24.

© 2024 Daly B et al. *JAMA Network Open.*

eMethods. Bloomberg New Economy International Cancer Coalition–Patient Centricity Survey

We are hoping to learn about the adoption of different **remote engagement and data collection approaches** for clinical trials. The survey will take **~15-20 mins**. Your time and insights are greatly appreciated.

Q1. Which organization do you represent? *(Text answer)*

Q2. From the following list of remote engagement and data collection approaches, please rank the **top 3** most impactful for **patient centricity** in the **short-term (next 1-3 years) (1=most impactful)** *(Rank top 3)*

- Digitally-enabled recruitment (e.g., patient identification, social media platforms to identify patients, etc.)
- Digitally-enabled enrollment (e.g., eConsent)
- Patient engagement dashboard to facilitate day-to-day trial participation and adherence (e.g., digital patient schedule, patient reimbursement tracking, digital assessment, and dose reporting)
- Telemedicine visits
- Shipment of medicines to patients' homes
- Laboratory collection near patients or engagement of mobile services
- Imaging at sites near patients or engagement of mobile services
- Remote monitoring (e.g., at-home pulse oximetry)
- Mobile nursing visits to enable discrete at-home monitoring (e.g., EKG)
- Visits in local physician networks
- eDiary and eCOA (e.g., ePRO) to capture notes on patient experience (incl. AEs) and determine efficacy of therapeutic interventions

Q3. From the following list of remote engagement and data collection approaches, please rank the **top 3 most impactful for patient centricity in the long-term (next 3+ years) (1=most impactful)** *(Rank top 3)*

- Digitally-enabled recruitment (e.g., patient identification, social media platforms to identify patients, etc.)
- Digitally-enabled enrollment (e.g., eConsent)

© 2024 Daly B et al. *JAMA Network Open.*

- Patient engagement dashboard to facilitate day-to-day trial participation and adherence (e.g., digital patient schedule, patient reimbursement tracking, digital assessment, and dose reporting)

  Telemedicine visits

- Shipment of medicines to patients' homes
- Laboratory collection near patients or engagement of mobile services
- Imaging at sites near patients or engagement of mobile services
- Remote monitoring (e.g., at-home pulse oximetry)
- Mobile nursing visits to enable discrete at-home monitoring (e.g., EKG)
- Visits in local physician networks
- eDiary and eCOA (e.g., ePRO) to capture notes on patient experience (incl. AEs) and determine efficacy of therapeutic interventions

Q4. From the following list of remote engagement and data collection approaches, please **estimate your organization's current adoption of approaches** for **registrational clinical trials across all therapeutic areas vs. registrational clinical trials in oncology** (e.g., what % of trials currently underway use the approach today)?

Please input number for percentage without percentage sign (e.g., 79 for 79%)

Input number for **registrational** clinical trials **across all therapeutic areas today**:

- Digitally-enabled recruitment (e.g., patient identification, social media platforms to identify patients, etc.)
- Digitally-enabled enrollment (e.g., eConsent)
- Patient engagement dashboard to facilitate day-to-day trial participation and adherence (e.g., digital patient schedule, patient reimbursement tracking, digital assessment, and dose reporting)
- Telemedicine visits
- Shipment of medicines to patients' homes
- Laboratory collection near patients or engagement of mobile services
- Imaging at sites near patients or engagement of mobile services
- Remote monitoring (e.g., at-home pulse oximetry)
- Mobile nursing visits to enable discrete at-home monitoring (e.g., EKG)
- Visits in local physician networks

- eDiary and eCOA (e.g., ePRO) to capture notes on patient experience (incl. AEs) and determine efficacy of therapeutic interventions

Input number for **registrational** clinical trials **in oncology today**:

- Digitally-enabled recruitment (e.g., patient identification, social media platforms to identify patients, etc.)
- Digitally-enabled enrollment (e.g., eConsent)
- Patient engagement dashboard to facilitate day-to-day trial participation and adherence (e.g., digital patient schedule, patient reimbursement tracking, digital assessment, and dose reporting)
- Telemedicine visits
- Shipment of medicines to patients' homes
- Laboratory collection near patients or engagement of mobile services
- Imaging at sites near patients or engagement of mobile services
- Remote monitoring (e.g., at-home pulse oximetry)
- Mobile nursing visits to enable discrete at-home monitoring (e.g., EKG)
- Visits in local physician networks
- eDiary and eCOA (e.g., ePRO) to capture notes on patient experience (incl. AEs) and determine efficacy of therapeutic interventions

Q5. From the following list of remote engagement and data collection approaches, what **adoption rate for registrational clinical trials in oncology** should the **industry aim to achieve in 5 years?**

Please input number for percentage without percentage sign (e.g., 79 for 79%) Input number for **registrational** clinical trials **in oncology in 5 years**:

- Digitally-enabled recruitment (e.g., patient identification, social media platforms to identify patients, etc.)
- Digitally-enabled enrollment (e.g., eConsent)
- Patient engagement dashboard to facilitate day-to-day trial participation and adherence (e.g., digital patient schedule, patient reimbursement tracking, digital assessment, and dose reporting)
- Telemedicine visits
- Shipment of medicines to patients' homes

- Laboratory collection near patients or engagement of mobile services
- Imaging at sites near patients or engagement of mobile services
- Remote monitoring (e.g., at-home pulse oximetry)
- Mobile nursing visits to enable discrete at-home monitoring (e.g., EKG)
- Visits in local physician networks
- eDiary and eCOA (e.g., ePRO) to capture notes on patient experience (incl. AEs) and determine efficacy of therapeutic interventions

Q6. What has **changed in the environment** to drive your organization to increase adoption of remote engagement and data collection approaches? *(Please select all that are relevant)*

- Changing expectations on heels of COVID-19 pandemic and its associated disruptions to clinical trials
- Competition from other organizations utilizing patient centric approaches
- Competition for patients in indications under investigation
- Other stakeholders have encouraged/mandated adoption (e.g., regulatory bodies)
- Innovation of novel technical solutions have significantly improved the quality and accessibility of these approaches
- N/A: We have not seen increased adoption
- Other: _________________

Q7. What are your organization's **key objective(s)** for the adoption of remote engagement and data collection approaches? *(Please select and rank your top 3 choices)*

- Broaden patient access to clinical trials
- Accelerate clinical trial execution
- Speed up patient recruitment/enrollment
- Reduce costs
- Improve data quality
- Increase diversity of patients participating in clinical trials
- Reduce patient dropout rate
- Other: _____________

© 2024 Daly B et al. *JAMA Network Open.*

Q8. How do you currently track the integrated **end-to-end experience** that patients are having in your trials? (e.g., TransCelerate's Study Participant Feedback Questionnaire (SPFQ), a protocol patient burden index etc.) *(Open response question)*

© 2024 Daly B et al. *JAMA Network Open.*
