# Supplementary material for: Remote Monitoring and Data Collection for Decentralized Clinical Trials
Source: JAMA Netw Open. 2024 Apr 12;7(4):e246228. doi: 10.1001/jamanetworkopen.2024.6228 (PMC11015350; doi:10.1001/jamanetworkopen.2024.6228)
Supplement: Supplement 2. — Data Sharing Statement [file jamanetwopen-e246228-s002.pdf]

Data Sharing Statement

Daly. Remote Monitoring and Data Collection for Decentralized Clinical Trials. *JAMA Netw Open*. Published April 12, 2024. doi:10.1001/jamanetworkopen.2024.6228

Data
Data available: Yes
Data types: Deidentified participant data
How to access data: dalyr1@mskcc.org
When available: With publication

Supporting Documents
Document types: None

Additional Information
Who can access the data: Researchers whose proposed use of the data has been approved
Types of analyses: For any purpose
Mechanisms of data availability: With investigator support
